# Diagnostic Accuracy of Preoperative Magnetic Resonance Imaging for Detecting Cervical Stromal Invasion in Endometrial Cancer: A Prospective Observational Study

**DOI:** 10.7759/cureus.107206

**Published:** 2026-04-16

**Authors:** Raghu Rami Reddy S, Yogesh Kheni, Kalla B Muralidhar, Joju Antony Sebastian

**Affiliations:** 1 Surgical Oncology, Pi Health Cancer Hospital, Hyderabad, IND; 2 Surgical Oncology, Starlit Cancer Center, Ahmedabad, IND; 3 Surgical Oncology, Mahatma Gandhi Cancer Hospital and Research Institute, Visakhapatnam, IND; 4 Surgical Oncology, Amala Institute of Medical Sciences, Kerala, IND

**Keywords:** cervical stromal invasion, endometrial cancer, histopathological correlation, magnetic resonance imaging, preoperative staging

## Abstract

Background

Accurate preoperative assessment of endometrial cancer is essential for optimal surgical planning and prognostication. Cervical stromal invasion upstages the disease to stage II and often necessitates a more radical surgical approach. Magnetic resonance imaging (MRI) is considered the imaging modality of choice for local staging due to its superior soft-tissue contrast and multiplanar capability.

Objective

To evaluate the diagnostic accuracy of preoperative contrast-enhanced MRI in detecting cervical stromal invasion in patients with endometrial cancer, using postoperative histopathological examination as the reference standard.

Methods

This prospective observational study was conducted at a tertiary care center from June 2020 to October 2021. A total of 30 patients with histologically confirmed stage I or stage II endometrial carcinoma underwent preoperative contrast-enhanced pelvic MRI. MRI findings were compared with postoperative histopathological results. Diagnostic performance was assessed using sensitivity, specificity, positive predictive value (PPV), negative predictive value (NPV), diagnostic accuracy, and Youden’s J index.

Results

The mean age of the study population was 59 years, and most patients were postmenopausal with early-stage disease. Cervical stromal invasion was identified in 4 patients (13.3%) on MRI and in 5 patients (16.7%) on histopathological examination. MRI demonstrated a sensitivity of 80% and specificity of 100%, with a PPV of 100% and NPV of 96.2%. The overall diagnostic accuracy was 96.77%, and Youden’s J index was 0.8.

Conclusion

Preoperative contrast-enhanced MRI demonstrates high diagnostic accuracy and excellent specificity in detecting cervical stromal invasion in endometrial cancer. It is a reliable modality for preoperative staging and may aid in guiding surgical management. Further multicenter studies with larger sample sizes are required to validate these findings.

## Introduction

Endometrial cancer is one of the most frequently diagnosed gynecologic malignancies worldwide and ranks among the leading cancers affecting women, with approximately 320,000 new cases reported each year [1]. Although the incidence in India remains lower than that observed in many Western countries, the overall burden of the disease has been gradually increasing [2]. The estimated lifetime risk of developing endometrial cancer by the age of 75 years varies between about 0.6% in developing regions and 1.6% in more industrialized nations [3]. The median age at diagnosis is around 61 years; however, nearly one-fifth of cases occur in premenopausal women, and roughly 5% are diagnosed in women younger than 40 years [2].

The precise etiology of endometrial cancer remains unclear, but prolonged unopposed estrogen exposure is considered the most significant risk factor. Conditions such as early menarche, late menopause, obesity, nulliparity, hypertension, diabetes mellitus, estrogen replacement therapy, and tamoxifen use contribute to increased estrogenic stimulation and heightened disease risk [4,5]. Abnormal uterine bleeding is the most common presenting symptom, reported in 80-90% of patients, while less frequent presentations include vaginal discharge, abnormal Papanicolaou smear findings, or incidentally detected endometrial thickening on ultrasonography [6]. Transvaginal ultrasonography is commonly used as the initial imaging modality in patients presenting with abnormal uterine bleeding. Parameters such as endometrial thickness and uterine artery Doppler indices may assist in preliminary risk stratification; however, ultrasound has limitations in accurately assessing myometrial invasion and cervical stromal involvement compared to MRI.

Histologically, endometrial carcinomas are broadly classified into two categories. Type I tumors, predominantly endometrioid adenocarcinomas, account for approximately 90% of cases and are estrogen-dependent, low-grade malignancies associated with a favorable prognosis. These tumors are graded according to the degree of cellular differentiation into grade 1 (well-differentiated), grade 2 (moderately differentiated), and grade 3 (poorly differentiated) categories [7]. In contrast, type II endometrial tumors, including serous carcinoma, clear cell carcinoma, and carcinosarcoma, are generally estrogen-independent and are more frequently observed in older women. These tumors typically exhibit more aggressive biological behavior and are associated with less favorable clinical outcomes. Both type II tumors and grade 3 endometrioid carcinomas are therefore considered high-grade malignancies [8].

Advances in molecular profiling have identified several genetic alterations associated with high-grade endometrial cancer, including mutations in p53, HER2/neu, and phosphatase and tensin homolog (PTEN). Overexpression of mutant p53 has been linked to non-endometrioid histology, advanced disease stage, and reduced survival [9,10]. In contrast, biomarkers such as p21 expression, microsatellite instability, and PTEN mutations have been associated with improved prognosis [11,12]. HER2/neu overexpression has been correlated with adverse outcomes in endometrial, breast, and ovarian cancers [13].

The prognosis of endometrial cancer is determined by multiple clinicopathological factors, such as the stage of the disease at presentation, tumor grade, depth of myometrial invasion, presence of lymphovascular space invasion, and involvement of regional lymph nodes [14]. Tumors with high histological grade and those demonstrating myometrial invasion greater than 50% have a higher likelihood of pelvic and para-aortic lymph node metastasis and are associated with less favorable survival outcomes [14]. Due to the early onset of symptoms, nearly 75% of patients are diagnosed at stage I disease. The reported 5-year survival rates are approximately 85% for stage I, 70% for stage II, 50% for stage III, and 18% for stage IV disease [15].

Magnetic resonance imaging (MRI) is regarded as the most accurate imaging modality for preoperative local staging of endometrial cancer due to its superior soft-tissue contrast and multiplanar imaging capability. On MRI, endometrial carcinoma typically appears hypo- to isointense on T1-weighted images and demonstrates intermediate signal intensity on T2-weighted images, with reduced enhancement compared to normal myometrium on dynamic contrast-enhanced sequences [16]. The reported overall accuracy of MRI for endometrial cancer staging ranges from 83% to 92% [16].

Dynamic contrast-enhanced MRI significantly improves the assessment of myometrial invasion. Pooled analyses have shown a sensitivity and specificity of 81% and 72%, respectively, for contrast-enhanced MRI, compared with 87% and 58% for T2-weighted imaging alone. When combined, T2-weighted and contrast-enhanced sequences achieve diagnostic accuracy of up to 98% for detecting myometrial invasion [16]. Evaluation of cervical stromal involvement is particularly critical, as it upgrades the disease to FIGO stage II and frequently alters surgical management. Normal cervical stroma demonstrates low signal intensity on T2-weighted images, whereas tumor infiltration appears as intermediate signal intensity. Dynamic contrast-enhanced MRI further enhances detection, with reported sensitivity, specificity, and diagnostic accuracy approaching 100% for cervical stromal invasion [16].In addition to staging, MRI plays an important role in differentiating endometrial carcinoma from other uterine malignancies, such as uterine sarcomas, which may present with overlapping clinical features but require different management strategies [17].

Considering the impact of cervical stromal involvement on both prognosis and management, accurate assessment prior to surgery is vital for guiding treatment decisions. This study was designed to determine the diagnostic performance of contrast-enhanced MRI in predicting cervical stromal invasion in patients with endometrial carcinoma, using postoperative histopathological findings as the reference standard.

## Materials and methods

Study design and ethical considerations

This prospective observational study was carried out at Basavatarakam Indo-American Cancer Hospital and Research Institute, Hyderabad, from June 2020 to October 2021. The study protocol received approval from the Institutional Ethics Review Board and was conducted in accordance with the principles outlined in the Declaration of Helsinki. Written informed consent was obtained from all participants prior to their inclusion in the study.

Study Population

A total of 30 patients with histologically confirmed stage I or stage II endometrial carcinoma were enrolled. Patients with stage III or IV disease, those who had received prior treatment for endometrial cancer, individuals with contraindications to magnetic resonance imaging (MRI), and patients who declined consent were excluded from the study.

All eligible patients presenting during the study period who met the inclusion criteria were consecutively included to minimize selection bias.

Sample Size

The sample size for the present study was calculated using Cochran’s formula for estimating sample size. Based on the expected prevalence and feasibility of patient recruitment during the study period, a total sample size of 30 patients was considered adequate to evaluate the diagnostic performance of MRI in detecting cervical stromal invasion in endometrial cancer.

The relatively small sample size was primarily due to the limited study duration and the low incidence of eligible cases at a single center. This has been acknowledged as a limitation.

MRI technique and image interpretation

All enrolled patients underwent preoperative pelvic MRI as part of the routine staging workup. Participants were instructed to fast for four to six hours prior to the examination and to empty their bladder immediately before imaging to maintain optimal visualization of pelvic structures. MRI examinations were performed using a 1.5-Tesla scanner with a phased-array pelvic coil.

The imaging protocol included T2-weighted sequences acquired in axial, axial-oblique, and sagittal planes, with axial-oblique images oriented perpendicular to the endometrial cavity. Native T1-weighted sagittal images were obtained, followed by fat-suppressed T1-weighted sequences in the axial-oblique and sagittal planes both before and four minutes after intravenous administration of a gadolinium-based contrast agent at a dose of 0.1 mmol/kg body weight. Contrast administration was avoided in patients with renal dysfunction, defined as a glomerular filtration rate (GFR) <30 mL/min.

All MRI scans were interpreted by an experienced radiologist who was aware of the clinical diagnosis but remained blinded to the postoperative histopathological results. Preservation of the low-signal-intensity junctional zone was interpreted as tumor limited to the endometrium, whereas disruption of this zone indicated myometrial invasion.

Cervical stromal invasion on MRI was defined as disruption of the normal low-signal-intensity cervical stroma on T2-weighted images with replacement by intermediate signal intensity tumor tissue, with or without abnormal enhancement on contrast-enhanced sequences.

Assessment of cervical involvement was performed by evaluating infiltration of the endocervical glands and cervical stroma, and radiologic staging was assigned in accordance with the FIGO staging system for endometrial cancer (2009) established by the International Federation of Gynecology and Obstetrics [18].

Surgical management and histopathological evaluation

All patients underwent surgical staging, including extrafascial hysterectomy with bilateral salpingo-oophorectomy and lymph node assessment. Patients with MRI evidence of cervical stromal involvement underwent type II radical hysterectomy.

Surgical specimens were sectioned in the coronal plane, and both gross and microscopic examinations were performed to assess cervical stromal invasion. Histopathological evaluation and final staging were conducted by a pathologist blinded to MRI findings, in accordance with the FIGO classification. MRI findings were subsequently compared with histopathological results, which served as the reference standard.

Statistical analysis

Statistical analysis was performed using the Statistical Package for the Social Sciences (SPSS) software version 25.0 (IBM Corp., Armonk, NY, US). The diagnostic performance of preoperative MRI in detecting cervical stromal invasion was evaluated by calculating sensitivity, specificity, positive predictive value (PPV), negative predictive value (NPV), diagnostic accuracy, and Youden’s J index using standard statistical formulas.

Sensitivity was calculated as the proportion of true positive (TP) cases among all patients with cervical stromal invasion or (TP / (TP + FN)), where FN represents false negative. Specificity was calculated as the proportion of true negative (TN) cases among patients without cervical stromal invasion (TN / (TN + FP)), where FP represents false positive. The positive predictive value (PPV) was calculated as (TP / (TP + FP)), and the negative predictive value (NPV) was calculated as (TN / (TN + FN)). Diagnostic accuracy was calculated as the proportion of correctly classified cases among the total number of cases ((TP + TN) / (TP + FP + TN + FN)).

Youden’s J index was calculated using the formula: Sensitivity + Specificity − 1.

## Results

Patient demographics and clinical characteristics

A total of 30 patients diagnosed with endometrial carcinoma were included in the study. The mean age of the patients was 59 years, with an age range of 43 to 78 years. The majority of patients (63.3%) were aged ≤60 years, while 36.7% were older than 60 years. Abnormal uterine bleeding was the predominant presenting symptom, reported in 96.7% of patients, whereas a small proportion presented with abdominal pain. The mean duration of symptoms was 2.7 months (standard deviation: 2.2 months).

Most patients were postmenopausal (96.7%). Comorbid conditions were present in 83.3% of patients, with hypertension being the most common, followed by diabetes mellitus. A small proportion of patients had no documented comorbidities. Assessment of body mass index (BMI) showed that the majority of patients were either overweight or obese, with half of the patients classified as obese and a substantial proportion as overweight (Table 1).

**Table 1:** Baseline demographic and clinical characteristics (N = 30)

Variable	Category	Frequency (n)	Percentage (%)
Age Group (years)	≤60	19	63.3%
	>60	11	36.7%
Mean Age (years)	59 (Range: 43–78)	-	-
Presenting Symptoms	Abnormal uterine bleeding	29	96.7
	Abdominal pain	1	3.3
Menopausal Status	Postmenopausal	29	96.7
	Premenopausal	1	3.3
Comorbidities	None	5	16.7
	Hypertension	22	73.3
	Diabetes	11	36.7
	Hypothyroidism	1	3.3
	Asthma	1	3.3
Body Mass Index (BMI)	Normal (18.5 – 24.9)	2	6.7
	Overweight (25.0 – 29.9)	13	43.3
	Obese (30 and above)	15	50

MRI findings and radiological staging

Preoperative contrast-enhanced MRI of the pelvis was performed in all patients. Cervical stromal involvement was identified on MRI in 13.3% of patients, while the majority showed no evidence of cervical involvement. Based on MRI staging, most patients were classified as FIGO stage IA, followed by stage IB and stage II disease (Table 2).

**Table 2:** MRI findings and radiological staging MRI: magnetic resonance imaging

Variable	Category	Frequency (n)	Percentage (%)
Cervical Stromal Involvement	Present	4	13.3
	Absent	26	86.7
	Total	30	100
Final Pathological Staging	IA	21	70.0
	IB	5	16.7
	II	4	13.3
	Total	30	100

Histopathological findings

Histopathological examination revealed that endometrioid adenocarcinoma was the most common histological subtype, accounting for the majority of cases, while a small proportion had serous carcinoma. Among endometrioid tumors, grade 1 tumors were most frequent, followed by grade 2 and grade 3 tumors. Cervical stromal involvement on histopathological examination was present in 16.7% of patients and absent in the remaining cases. Final pathological staging showed that most patients had stage IA disease, followed by stage IB and stage II disease (Table 3).

**Table 3:** Histopathological findings and final staging

Variable	Category	Frequency (n)	Percentage (%)
Cervical Stromal Involvement	Present	5	16.7
	Absent	25	83.3
	Total	30	100%
Final Pathological Staging	IA	21	70.0
	IB	4	13.3
	II	5	16.7
	Total	30	100%

Surgical management

Based on preoperative MRI findings, 26 patients diagnosed with stage I disease underwent laparoscopic extrafascial hysterectomy with bilateral salpingo-oophorectomy. Four patients with MRI evidence of stage II disease underwent laparoscopic type II radical hysterectomy. Surgical procedures were completed successfully in all patients without intraoperative staging discrepancies (Table 4).

**Table 4:** Surgical procedures performed EFH: extrafascial hysterectomy; BSO: bilateral salpingo-oophorectomy; RH: radical hysterectomy; MRI: magnetic resonance imaging

Surgery	Frequency	Percentage
EFH + BSO	26	86.6
Type II RH	4	13.3
Total	30	100

Diagnostic accuracy of MRI for cervical stromal invasion

Correlation between preoperative MRI findings and histopathological examination showed that MRI correctly identified cervical stromal invasion in most cases. One case of cervical stromal invasion was not detected on MRI, resulting in a false-negative finding, while no false-positive cases were observed (Table 5).

**Table 5:** Correlation between MRI and histopathology MRI: magnetic resonance imaging

Preoperative MRI Cervical Involvement	Histopathology Present	Histopathology Absent
Present	4 (80%)	0 (0%)
Absent	1 (20%)	25 (100%)
Total	5 (100%)	25 (100%)

Preoperative MRI demonstrated a sensitivity of 80% and specificity of 100% for detecting cervical stromal invasion. The positive predictive value was 100%, and the negative predictive value was 96.2%. The overall diagnostic accuracy was 96.77%. Youden’s J index was 0.8, indicating good diagnostic performance (Table 6).

**Table 6:** Diagnostic performance of MRI MRI: magnetic resonance imaging

Diagnostic Parameter	Value (%)	95% Confidence Interval
Sensitivity	80%	8.36% - 99.49%
Specificity	100%	86.77% - 100%
False Positive Rate	0%	0% - 13.23%
False Negative Rate	20%	0.51% - 71.64%
Diagnostic Accuracy	96.77%	83.30% - 99.92%
Youden’s J Index	0.80	-

## Discussion

Accurate preoperative staging is a cornerstone in the management of endometrial cancer, as it directly influences surgical planning and prognosis. In the revised 2009 International Federation of Gynecology and Obstetrics (FIGO) staging system, cervical stromal invasion defines stage II disease, signifying progression from disease confined to the uterine corpus to locally advanced cancer [18]. While patients with disease limited to the endometrium generally have favorable outcomes, the presence of cervical stromal involvement is associated with reduced survival and an increased risk of extrauterine spread [19].

MRI is widely regarded as the preferred imaging modality for preoperative local staging of endometrial cancer due to its superior soft-tissue contrast resolution and multiplanar capability [20]. Accurate evaluation of cervical stromal invasion is particularly important, as it has direct implications for surgical decision-making, including the need for more radical procedures.

In this prospective study, we evaluated the diagnostic performance of contrast-enhanced MRI in detecting cervical stromal invasion, using histopathological examination as the reference standard. The demographic profile of our cohort was consistent with known epidemiological patterns, with most patients being postmenopausal and a high prevalence of overweight and obesity, which are established risk factors for endometrial carcinoma. The majority of patients in our study were diagnosed at an early stage, reflecting the typical clinical presentation of endometrial cancer. Endometrioid adenocarcinoma was the predominant histological subtype, consistent with existing literature.

Preoperative MRI identified cervical stromal involvement in a small proportion of patients. Histopathological examination confirmed one additional case, resulting in a single false-negative MRI finding. No false-positive cases were observed. The diagnostic accuracy of MRI in our study was high, with excellent specificity and strong overall performance. These findings are comparable to previously published studies, including those by Manfredi et al. and Santhanam Sampath et al., which have reported similar diagnostic accuracy [21,22].

The sensitivity observed in our study was within the range reported in the literature. Variability in sensitivity across studies may be attributed to differences in imaging protocols, observer experience, and study populations. The high specificity observed reinforces the reliability of MRI in confirming cervical stromal involvement when present. The high positive predictive value and negative predictive value observed in this study further support the clinical utility of MRI in preoperative assessment. In particular, the high negative predictive value suggests that MRI may be useful in excluding cervical stromal invasion, thereby helping to avoid unnecessary radical surgical procedures in early-stage disease.

The strengths of this study include its prospective design and the use of histopathological examination as the reference standard, with blinded assessment, which reduces potential bias. However, certain limitations should be acknowledged. The study was conducted at a single center with a relatively small sample size, which may limit the generalizability of the findings. The small sample size was influenced by the limited study duration and the low incidence of eligible cases during the study period. Additionally, interobserver variability in MRI interpretation was not assessed, which may influence diagnostic performance.

Future studies with larger sample sizes and multicenter participation are warranted to validate these findings and further establish the role of MRI in preoperative staging. Overall, the findings of this study support the role of contrast-enhanced MRI as a reliable imaging modality for the preoperative evaluation of cervical stromal invasion in endometrial cancer, with the potential to guide appropriate surgical management.

## Conclusions

Contrast-enhanced MRI demonstrated high diagnostic accuracy and excellent specificity in the preoperative detection of cervical stromal invasion in patients with endometrial cancer. The strong agreement between MRI findings and histopathological examinations supports its role as a reliable imaging modality for local staging.

Accurate identification of cervical stromal involvement is essential for appropriate surgical planning and may help in selecting patients who require more radical surgical approaches, while avoiding overtreatment in early-stage disease. However, the findings of this study should be interpreted in the context of its limitations, including the relatively small sample size and single-center design. Further large-scale, multicenter studies are warranted to validate these results and strengthen the evidence base for the routine use of MRI in preoperative staging of endometrial carcinoma.

## References

[1] Ferlay J, Steliarova-Foucher E, Lortet-Tieulent J (2013). Cancer incidence and mortality patterns in Europe: estimates for 40 countries in 2012. Eur J Cancer.

[2] Maheshwari A, Kumar N, Mahantshetty U (2016). Gynecological cancers: a summary of published Indian data. South Asian J Cancer.

[3] Jemal A, Siegel R, Ward E, Hao Y, Xu J, Murray T, Thun MJ (2008). Cancer statistics, 2008. CA Cancer J Clin.

[4] (2014). Berek & Hacker’s Gynecologic Oncology. 6th Edition. https://books.google.co.in/books?hl=en&lr=&id=bA3ODcFV-5oC&oi=fnd&pg=PR5&dq=Berek+JS,+Hacker%27s+NF.+Berek+and+Hacker%E2%80%99s+Gynecologic+Oncology.+6th+Edition.+Philadelphia,+Pa%3B+London:+Lippincott+Williams.

[5] McPherson CP, Sellers TA, Potter JD, Bostick RM, Folsom AR (1996). Reproductive factors and risk of endometrial cancer. The Iowa Women's Health Study. Am J Epidemiol.

[6] Burbos N, Musonda P, Duncan TJ, Crocker SG, Morris EP, Nieto JJ (2011). Estimating the risk of endometrial cancer in symptomatic postmenopausal women: a novel clinical prediction model based on patients' characteristics. Int J Gynecol Cancer.

[7] Wright JD, Barrena Medel NI, Sehouli J, Fujiwara K, Herzog TJ (2012). Contemporary management of endometrial cancer. Lancet.

[8] Tirumani SH, Shanbhogue AK, Prasad SR (2013). Current concepts in the diagnosis and management of endometrial and cervical carcinomas. Radiol Clin North Am.

[9] Fadare O, Gwin K, Desouki MM (2013). The clinicopathologic significance of p53 and BAF-250a (ARID1A) expression in clear cell carcinoma of the endometrium. Mod Pathol.

[10] Attias-Geva Z, Bentov I, Kidron D (2012). p53 Regulates insulin-like growth factor-I receptor gene expression in uterine serous carcinoma and predicts responsiveness to an insulin-like growth factor-I receptor-directed targeted therapy. Eur J Cancer.

[11] Steinbakk A, Malpica A, Slewa A (2011). Biomarkers and microsatellite instability analysis of curettings can predict the behavior of FIGO stage I endometrial endometrioid adenocarcinoma. Mod Pathol.

[12] Minaguchi T, Yoshikawa H, Oda K (2001). PTEN mutation located only outside exons 5, 6, and 7 is an independent predictor of favorable survival in endometrial carcinomas. Clin Cancer Res.

[13] Smith-Bindman R, Kerlikowske K, Feldstein VA (1998). Endovaginal ultrasound to exclude endometrial cancer and other endometrial abnormalities. JAMA.

[14] Epstein E, Blomqvist L (2014). Imaging in endometrial cancer. Best Pract Res Clin Obstet Gynaecol.

[15] Faria SC, Sagebiel T, Balachandran A, Devine C, Lal C, Bhosale PR (2015). Imaging in endometrial carcinoma. Indian J Radiol Imaging.

[16] Beddy P, O'Neill AC, Yamamoto AK, Addley HC, Reinhold C, Sala E (2012). FIGO staging system for endometrial cancer: added benefits of MR imaging. Radiographics.

[17] Nguyen PN, Nguyen VT (2022). Endometrial thickness and uterine artery Doppler parameters as soft markers for prediction of endometrial cancer in postmenopausal bleeding women: a cross-sectional study at tertiary referral hospitals from Vietnam. Obstet Gynecol Sci.

[18] Abu-Rustum NR, Zhou Q, Iasonos A (2011). The revised 2009 FIGO staging system for endometrial cancer: should the 1988 FIGO stages IA and IB be altered?. Int J Gynecol Cancer.

[19] Yin XH, Jia HY, Shi M, Wu H, Li YM (2015). Magnetic resonance imaging for detection of depth of myometrial invasion and cervical invasion in patients with endometrial carcinoma. Int J Clin Exp Med.

[20] Zamani F, Goodarzi S, Hallaji F, Zamiri A, Deilami T, Malek M, Modarress Gilani M (2012). Diagnostic value of pelvic MRI for assessment of the depth of myometrial invasion and cervical involvement in endometrial cancer: comparison of new versus old FIGO staging. Iran J Radiol.

[21] Sampath S, Nema D, Agarwal R, Lele P (2017). Accuracy of MR imaging in endometrial cancer: our experience. Int J Reprod Contracept Obstet Gynecol.

[22] Manfredi R, Mirk P, Maresca G (2004). Local-regional staging of endometrial carcinoma: role of MR imaging in surgical planning. Radiology.

